# Prognostic value of the prognostic nutritional index in sepsis, pneumonia, and tuberculosis: a systematic review and meta-analysis

**DOI:** 10.3389/fmed.2026.1879397

**Published:** 2026-09-21

**Authors:** Zhiheng Fu, Wanpeng Wang

**Affiliations:** Weifang People’s Hospital, Shandong Second Medical University, Weifang, Shandong, China

**Keywords:** infectious diseases, meta-analysis, PNI, prognosis, prognostic nutritional index, systematic review

## Abstract

**Objective:**

To evaluate the prognostic value of the Prognostic Nutritional Index (PNI) in patients with infectious diseases, focusing on tuberculosis, pneumonia, and sepsis. By synthesizing extant evidence, we examine the association between baseline PNI and clinical outcomes to inform early risk stratification and nutritional monitoring. Additionally, we probe underlying pathophysiological mechanisms, offering prognostic insights.

**Methods:**

A systematic literature search was conducted across five databases, including PubMed, Web of Science, Embase, the Cochrane Library, and China National Knowledge Infrastructure (CNKI), from inception to June 2026. Following methodological appraisal via the Newcastle-Ottawa Scale, we investigated associations between PNI and mortality or unfavorable outcomes. PNI was uniformly calculated at baseline using the formula: PNI = serum albumin (g/L) + 5 × lymphocyte count (×10^9^/L). Meta-analysis, Egger's test, and sensitivity analysis were performed in R software (version 4.5.0).

**Results:**

From an initial pool of 1,383 relevant studies, 10 studies comprising 28,757 patients were included in the final analysis. Disease-stratified meta-analysis underscores PNI's prognostic association in infectious diseases. Notably, among sepsis patients, elevated baseline PNI is associated with a significantly attenuated mortality risk (hazard ratio [HR]: 0.63; 95% confidence interval [CI]: 0.44, 0.90). Similarly, in the pneumonia subgroup, a high PNI demonstrated a significant inverse association with mortality (HR: 0.47; 95% CI: 0.30, 0.73). Conversely, in the tuberculosis subgroup, the association between PNI and mortality did not reach statistical significance (HR: 0.38; 95% CI: 0.04, 3.82). Overall, higher PNI remained a significant protective factor against mortality or unfavorable clinical outcomes (pooled HR: 0.52; 95% CI: 0.39, 0.71; *P* < 0.0001), with leave-one-out sensitivity analyses confirming the stability of these findings. Neither Begg's test (*P* = 0.2219) nor Egger's test (*P* = 0.1009) provided statistical evidence of small-study effects, although the limited number of included studies reduces the power of these tests.

**Conclusions:**

Reduced baseline PNI correlates with heightened mortality in sepsis and pneumonia, yet its prognostic value in tuberculosis remains uncertain. Although PNI is a cost-effective clinical adjunct, the current evidence is too heterogeneous to support its universal use in infectious diseases. Addressing these gaps requires large-scale prospective investigations across a wider spectrum of diseases.

## 1 Introduction

Initially introduced by Mullen et al. (1979) to quantify the prognostic impact of malnutrition on gastrointestinal surgery outcomes, the Prognostic Nutritional Index (PNI) correlates suboptimal nutritional status with an increased risk of postoperative morbidity and mortality (1). The original formula relies on diverse parameters including serum albumin, triceps skinfold thickness, transferrin, and delayed-type hypersensitivity. Subsequent clinical validation by Buzby et al. established this framework as the Mullen/Buzby PNI (2). However, its computational complexity and reliance on numerous variables have historically limited its broad clinical implementation (3). Subsequently, Onodera et al. simplified the formula to: PNI = serum albumin (g/L) + 5 × lymphocyte count (×10^9^/L) (4). While extensively validated across oncology, surgery, cardiovascular disease, and autoimmune disorders, this simplified PNI has only recently gained traction as a prognostic tool within the realm of infectious diseases (5–8).

The prognostic value of the PNI derives from both the pathophysiological significance of its individual components and their synergistic systemic profile. Serum albumin declines through two primary pathways: organ-specific pathologies (e.g., cirrhosis, nephrotic syndrome) and systemic inflammation combined with malnutrition—the predominant driver in infectious diseases (9, 10). During severe infection, interleukin-6 (IL-6) induces hypoalbuminemia by suppressing hepatocyte albumin transcription via the JAK/STAT3 pathway. Concurrently, this proinflammatory surge accelerates albumin catabolism and increases microvascular permeability, precipitating substantial protein extravasation (11).

Consequently, during infection, serum albumin transcends its role as a mere nutritional metric, serving as a robust surrogate for systemic inflammatory burden. Concurrently, peripheral lymphocyte counts quantify adaptive immune reserves (12). Infection-associated lymphopenia is multifactorial, driven by glucocorticoid-induced cellular redistribution, cytokine-mediated apoptosis, and malnutrition-attenuated lymphocyte proliferation (13). Therefore, a depressed PNI captures the concurrent collapse of both axes, signaling a vicious cycle of immunological exhaustion and nutritional depletion. Theoretically, this synergistic deficit provides biological plausibility for the prognostic relevance of PNI in infectious diseases.

Among the prognostic assessment tools currently available for infectious diseases, indices such as the Neutrophil-to-Lymphocyte Ratio (NLR), Monocyte-to-Lymphocyte Ratio (MLR), and Systemic Immune-Inflammation Index (SII) primarily reflect dysregulation along the inflammatory-immune axis but lack a nutritional dimension (14, 15). The Controlling Nutritional Status (CONUT) score incorporates serum albumin, total cholesterol, and lymphocyte counts, yet employs a categorical stratification approach and requires lipid profiling, which may limit its accessibility in resource-constrained settings. Established scoring systems, such as the Sequential Organ Failure Assessment (SOFA) and Acute Physiology and Chronic Health Evaluation II (APACHE II), provide robust quantification of organ dysfunction but fail to explicitly capture the host's nutritional reserve (16, 17). The Nutrition Risk in Critically Ill (NUTRIC) score and the modified Nutrition Risk in Critically Ill (mNUTRIC) score address this by integrating SOFA, APACHE II, age, comorbidities, and pre-intensive care unit (ICU) hospital stay, serving as reliable indicators of nutritional risk in critical care settings. With its simplified scoring criteria, the mNUTRIC score has also been validated to assess the prognosis of septic patients in general internal medicine wards, thereby enhancing its clinical practicality (18). Nevertheless, its routine application remains restricted, as it still mandates the collection of an extensive array of clinical parameters.

In contrast, the PNI is constructed solely from two routinely available clinical parameters and captures information related to both nutritional and immune status within a single continuous index. This combination of parsimony, multidimensional insight, and broad accessibility may provide a pragmatic advantage in certain clinical settings. However, whether this translates into incremental predictive value relative to NLR, MLR, SII, CONUT, or NUTRIC/mNUTRIC, as well as whether its prognostic relevance is consistent across different infectious conditions, remains to be rigorously validated.

To bridge these gaps, this systematic review and meta-analysis integrates available clinical evidence up to June 2026 across distinct infectious diseases. Our primary objective is to evaluate the differential prognostic utility of baseline PNI and to determine whether its prognostic association varies with distinct infectious diseases, thereby informing future risk-stratification research and guiding more targeted therapeutic strategies.

## 2 Methods

### 2.1 Literature search

From inception to June 2026, we systematically searched five electronic databases: PubMed, Web of Science, Embase, the Cochrane Library, and China National Knowledge Infrastructure (CNKI), to identify studies quantifying the prognostic utility of PNI across infectious diseases (19). The search terms were as follows: “((“infection*” [Mesh]) OR (((sepsis) OR (pneumonia)) OR (tuberculosis)) OR (hepatitis)) AND ((prognostic nutritional index [Title/Abstract]) OR (PNI))”. Additionally, the reference lists of all included studies were manually screened. There were no language or date restrictions for literature retrieval. Two investigators (ZHF and WPW) independently vetted the retrieved titles and abstracts, followed by a full-text review to determine final eligibility; any disagreements were resolved through discussion to reach a consensus. Search details are provided in Supplementary Table S1.

### 2.2 Inclusion and exclusion criteria

Inclusion criteria:

P: Patients diagnosed with infectious diseases, including but not limited to sepsis, community-acquired pneumonia (CAP), and tuberculosis.

E: The lowest category of baseline PNI, calculated using the formula: PNI = serum albumin (g/L) + 5 × lymphocyte count (×10^9^/L)

C: The highest category of baseline PNI

O: Mortality or unfavorable clinical outcomes assessed at the longest available follow-up.

S: Cohort studies.

Exclusion criteria: Non-original research (including letters, comments, editorials, abstracts, corrections, and replies); study protocols or unpublished manuscripts; single-arm studies or studies without a comparative design; studies from which hazard ratios (HRs) and corresponding 95% confidence intervals (CIs) for survival outcomes could not be directly extracted or calculated from the published data; and multiple publications arising from the same study population, in which case only the most comprehensive or most recent report was retained.

### 2.3 Data extraction

Two investigators (ZHF and WPW) independently extracted data, resolving any discrepancies via consensus. Data extracted from eligible studies encompassed first author's name, publication year, country (study location), study design, study period, study population, outcomes, sample size, patient sex, patient age, and PNI cut-off thresholds. For baseline variables that could not be retrieved, we explicitly reported the missingness in the baseline characteristics table and synthesized only observed outcomes. Preference was given to extracting multivariate-adjusted HRs (adjusting for variables such as age, SOFA, APACHE II, and the Simplified Acute Physiology Score II [SAPS II] scores, alongside therapeutic interventions).

### 2.4 Quality assessment

Included studies were evaluated via the Newcastle-Ottawa Scale (NOS) (20); scores of 7–9 were considered to be of high methodological quality (21). Two reviewers independently assessed the methodological quality of the included studies, resolving any discrepancies through consensus-oriented discussion. Detailed scoring data are presented in Supplementary Table S2.

### 2.5 Statistical analysis

Statistical analyses were performed using R software (version 4.5.0), and a two-tailed *P*-value < 0.05 was considered statistically significant. Pooled effect sizes were expressed as HRs for time-to-event survival and odds ratios (ORs) for dichotomous variables, both reported with a 95% CI. To account for anticipated clinical and methodological diversity, a random-effects model (DerSimonian-Laird) was applied across all metrics. Heterogeneity was quantified using Cochran's *Q* test and the I^2^ statistic; the Q test *P*-value < 0.10 or I^2^ > 50% indicated significant inconsistency (22, 23). Pre-specified subgroup analyses were conducted by disease type. To evaluate the stability of pooled estimates, sensitivity analyses were performed for outcomes including more than two studies using the leave-one-out approach, thereby isolating the influence of individual studies (24).

## 3 Results

### 3.1 Literature search and study characteristics

The literature search process (Figure 1) identified 1,383 records across PubMed (*n* = 393), Web of Science (*n* = 73), Cochrane Library (*n* = 36), CNKI (*n* = 85), and Embase (*n* = 796). Following deduplication, we screened the titles and abstracts of 1,116 identified records. Ten eligible studies comprising 11 independent cohorts (total *n* = 28,757) were synthesized in this meta-analysis (25–34). Although our initial search strategy broadly encompassed other infectious diseases such as hepatitis, studies related to these conditions were ultimately excluded during the full-text review phase. This was primarily due to the lack of extractable HRs and 95% CIs for survival outcomes. Table 1 summarizes the baseline characteristics of the included studies, which were published between 2022 and 2025.

**Figure 1:**
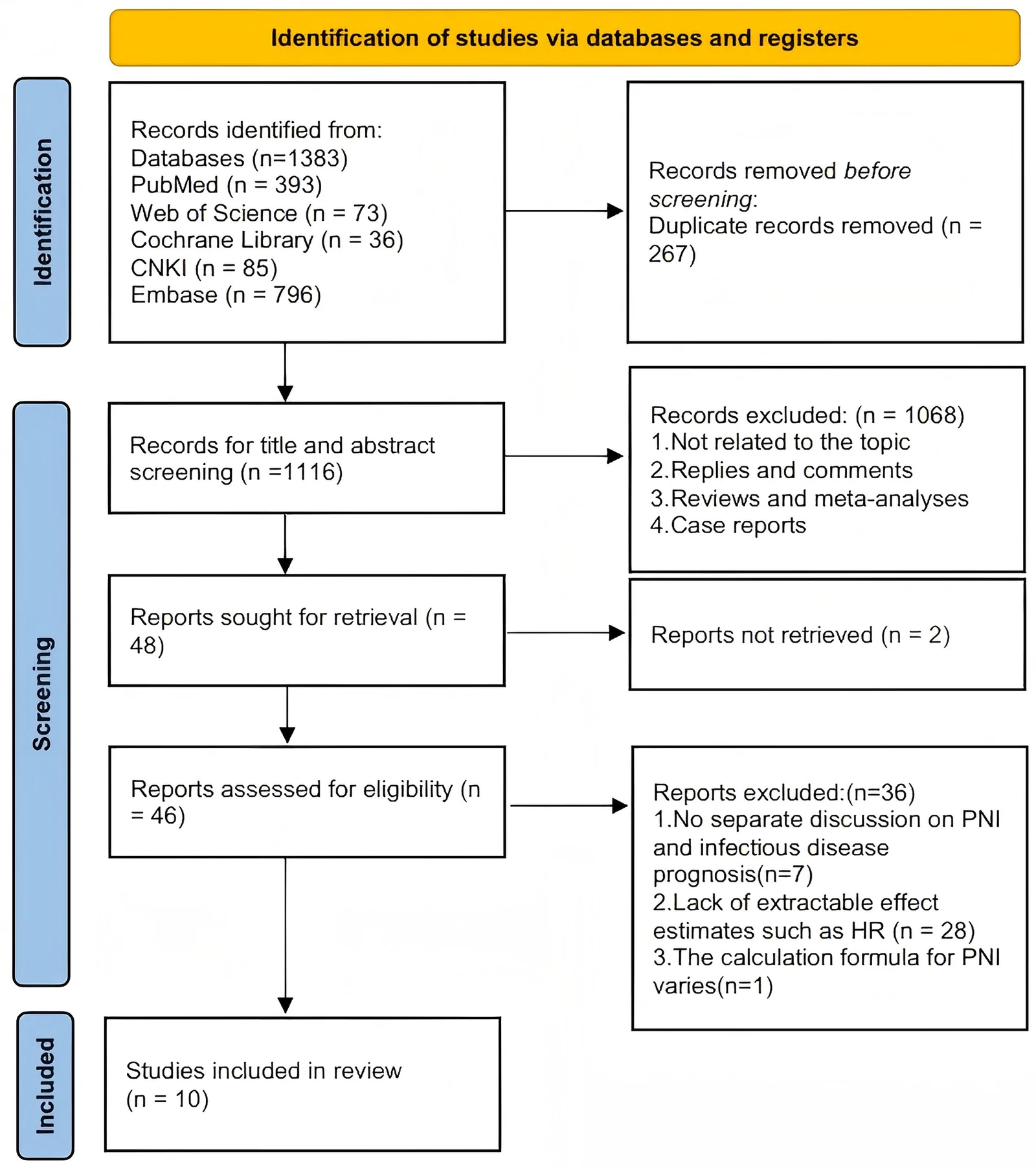
PRISMA flow diagram of study selection.

**Table 1:** Baseline characteristics of studies included in the meta-analysis.

Study (year)	Country	Study design	Target population	Sample size (*N*)	Male*(*n)	Mean/median age	Outcomes	Follow-up (months)	PNI cut-off	Adjusted HR (95% CI)
Fang et al. (2025) (25)	United States	Retrospective cohort	Sepsis	1649	993	NA	Mortality	2	30.0	0.47 (0.30–0.73)
Xie et al. (2025) (26)	United States	Retrospective cohort	Sepsis	5786	3262	64.3	Mortality	3	27.0	0.79 (0.71–0.88)
Baek et al. (2024) (27)	South Korea	Retrospective cohort	Sepsis	9763	5683	NA	Mortality	1	35.0	0.53 (0.47–0.60)
Wu et al. (2022) (28)	United States	Retrospective cohort	Sepsis	2669	1483	66.2	Mortality	12	29.3	0.69 (0.61–0.78)
Ding et al. (2025) (29)	China	Retrospective cohort	Community-acquired pneumonia	635	332	58.3	Mortality	3	34.4	0.39 (0.25–0.63)
Xue et al. (2025) (30)	China	Retrospective cohort	Community-acquired pneumonia	639	334	NA	Mortality	3	43.0	0.51 (0.31–0.83)
Ji et al. (2025) (31)	United States	Retrospective cohort	Ventilator-associated pneumonia	1457	936	64.1	Mortality	3	31.3	0.64 (0.50–0.82)
Wang et al. (2024) (34)	United States	Retrospective cohort	Community-acquired pneumonia	1608	920	67.0	Mortality	3	31.1	0.36 (0.28–0.47)
Ji et al. (2025) (32)	China	Retrospective cohort	Tuberculosis	4027	2504	38.0	Unfavorable outcome	12	NA	1.02 (0.74–1.41)
Hu et al. (2024) (33)-Training	China	Retrospective cohort	Tuberculosis	255	178	43.5	Mortality	24	38.7	0.25 (0.11–0.6)
Hu et al. (2024) (33)-Validation	China	Retrospective cohort	Tuberculosis	269	NA	41.9	Mortality	24	38.7	0.19 (0.09–0.40)

Unfavorable outcome, including treatment failure and death.

### 3.2 Prognostic value of PNI for mortality or unfavorable outcome

We pooled data on mortality and unfavorable outcomes from 11 independent cohorts across 10 studies. The meta-analysis revealed that increased PNI levels were significantly associated with a decreased risk of mortality and unfavorable clinical outcomes (HR: 0.52; 95% CI: 0.39–0.71; *P* < 0.001), although substantial inter-study heterogeneity was observed (I^2^ = 86.2%, *P* < 0.001) (Figure 2).

**Figure 2:**
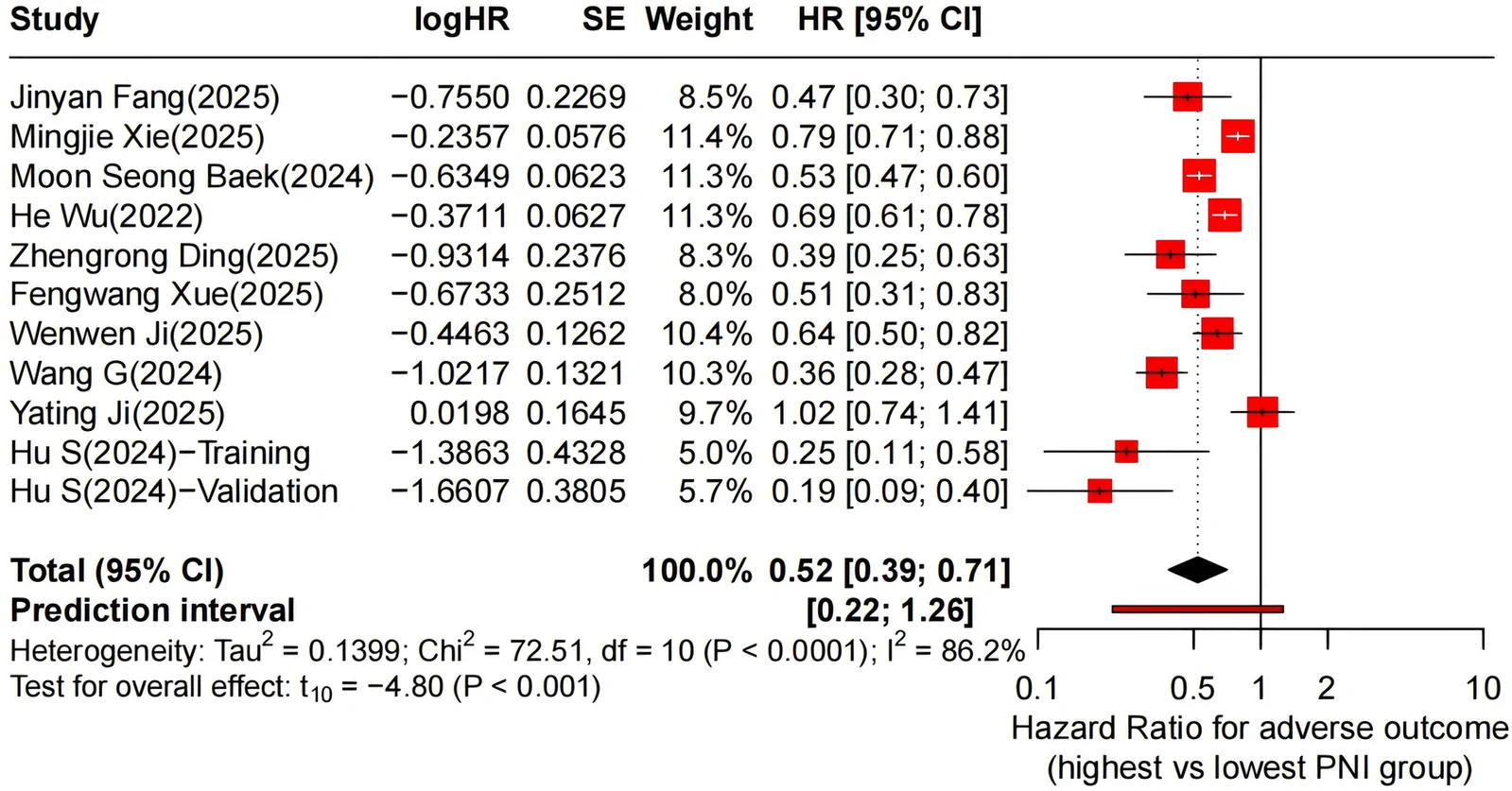
Forest plots of the prognostic value of PNI for mortality and unfavorable outcomes.

### 3.3 Subgroup analysis

Subgroup analyses by disease category demonstrated differences in the prognostic association of PNI across infectious diseases. The meta-analysis indicated that higher PNI levels were significantly associated with a decreased risk of sepsis-related mortality and unfavorable outcomes (4 studies; HR = 0.63, 95% CI: 0.44–0.90, *I*^2^ = 87.9%). The pneumonia subgroup (4 studies) exhibited a similar significant association (HR:0.47, 95% CI: 0.30, 0.73; I^2^ = 71.9%). Conversely, no significant association between PNI and poor prognosis was identified in the tuberculosis subgroup (2 studies comprising 3 analytical cohorts; HR:0.38, 95% CI: 0.04,3.82; I^2^ = 91.2%) (Figure 3).

**Figure 3:**
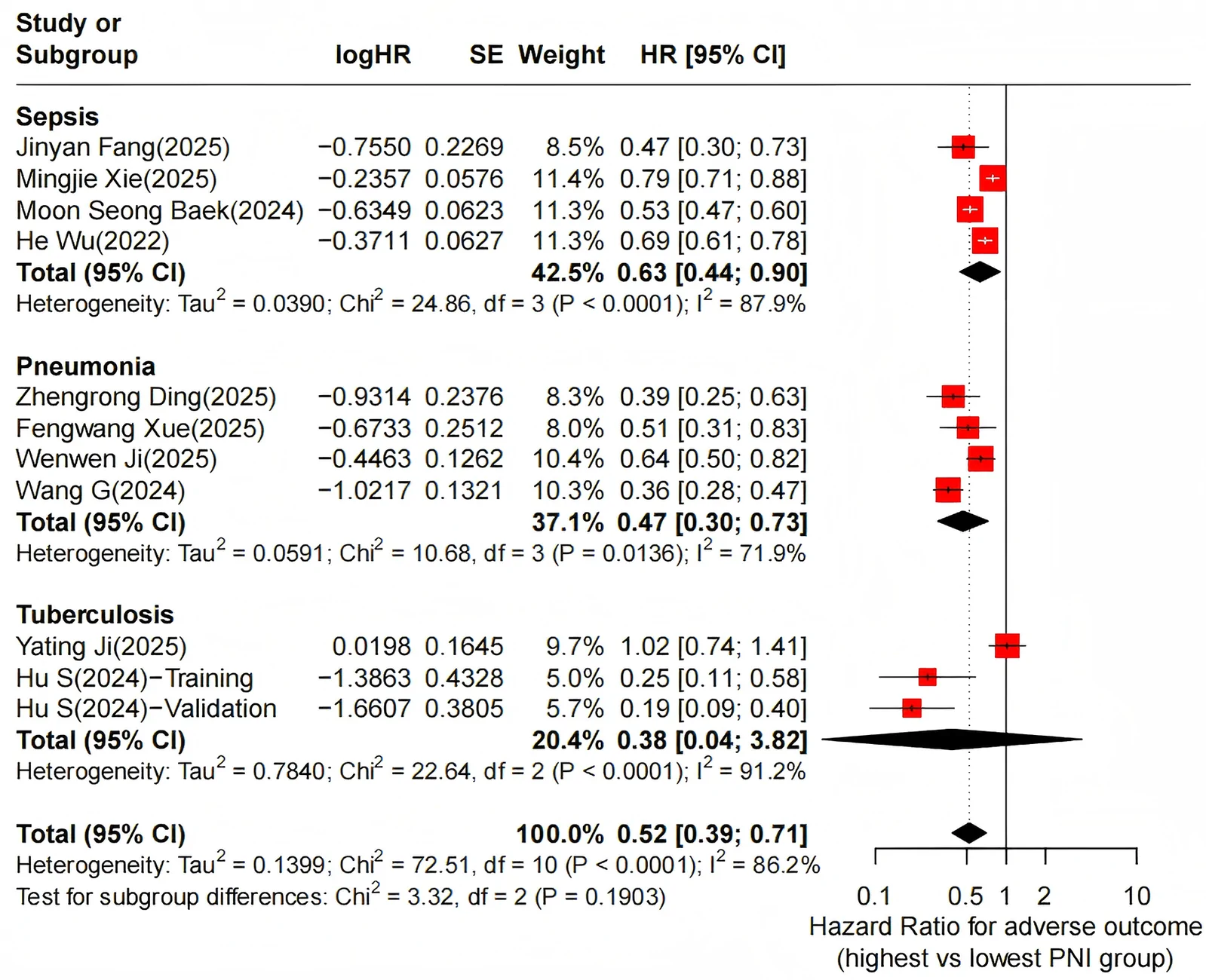
Forest plots of subgroup analyses by disease type (sepsis, pneumonia, tuberculosis).

### 3.4 Publication bias

Publication bias was assessed using Begg's rank correlation test and Egger's linear regression test. Neither Begg's nor Egger's test indicated statistically significant funnel plot asymmetry (*P* > 0.05) (Figure 4, Table 2).

**Figure 4:**
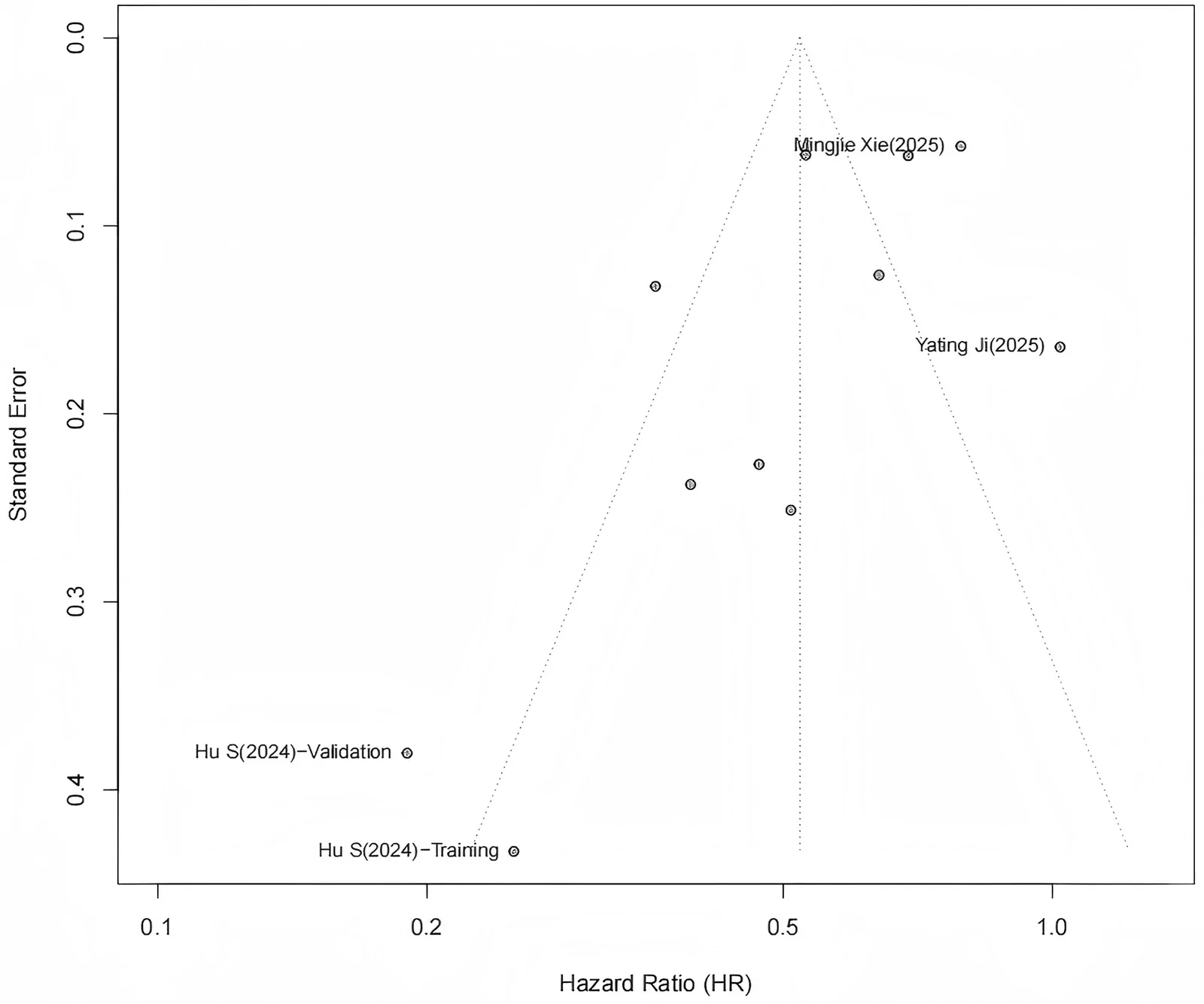
Funnel plot for visual assessment of publication bias across the included studies.

**Table 2:** Begg’s and egger’s test results for publication bias assessment.

Test	Statistic	*P*-value	Bias estimate (SE)
Begg's rank correlation test	z = −1.25	0.2219	−16.0000 (12.8452)
Egger's linear regression test	t = −1.83 (df = 9)	0.1009	−2.3880 (0.1347)

### 3.5 Sensitivity analysis

A leave-one-out sensitivity analysis showed that the omission of any single study did not materially alter the pooled estimates. The results showed that no single study materially changed the direction or statistical significance of the pooled estimate (Figure 5).

**Figure 5:**
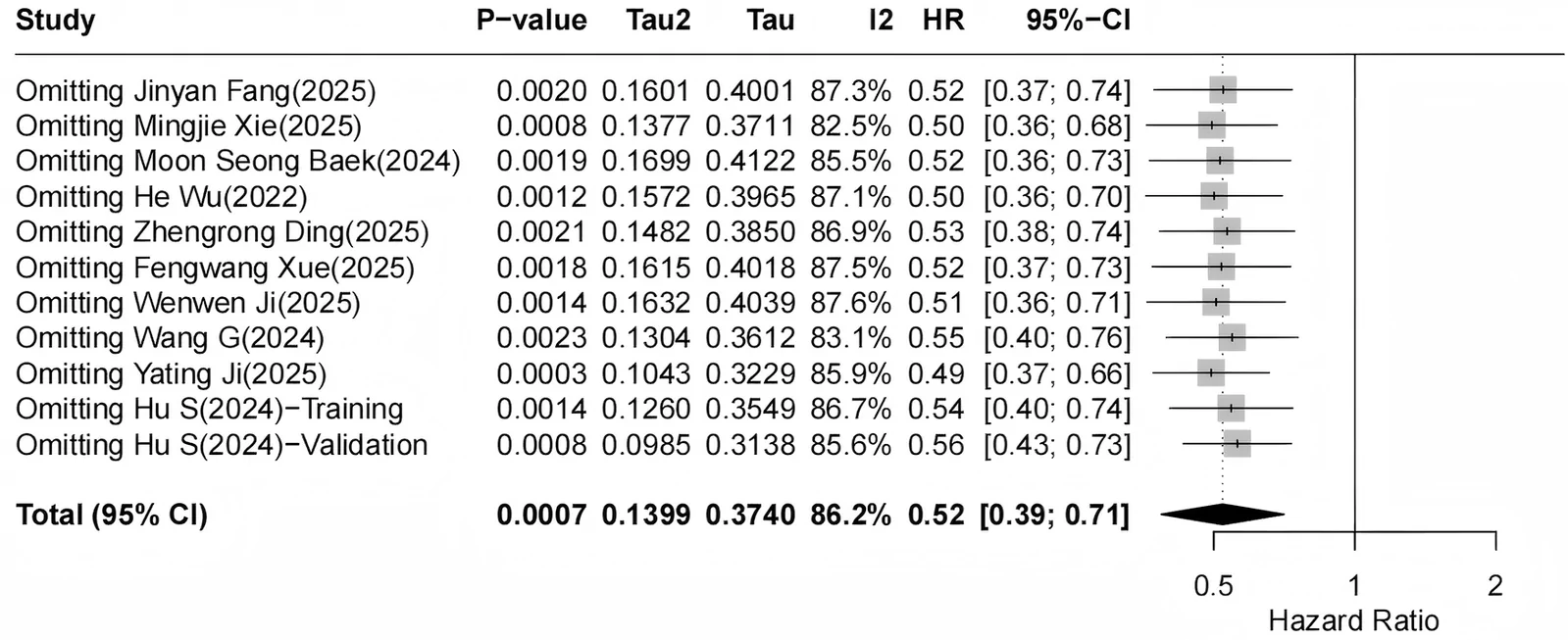
Leave-one-out sensitivity analysis of the prognostic value of PNI for mortality and unfavorable outcomes.

### 3.6 Meta-regression

In univariable meta-regression, none of the pre-specified moderators significantly explained the between-study heterogeneity. The PNI cutoff value showed a borderline association with the effect size (*β* = −0.0469, 95% CI: −0.102 to 0.008, *P* = 0.084), suggesting that differences in cutoff selection may partially contribute to the observed heterogeneity. Follow-up duration, country/region, and NOS score were not significantly associated with the effect size. We further performed an exploratory multivariable meta-regression including PNI cutoff value, follow-up duration, and NOS score. None of these covariates remained statistically significant in the multivariable model. Given the limited number of included effect estimates, all meta-regression analyses were interpreted as exploratory (Supplementary Table S3).

### 3.7 Stratified analysis

Studies were divided according to the median PNI cutoff value of 32.8. The association between higher PNI and lower risk of unfavorable outcomes remained directionally consistent in both the higher cutoff group (HR = 0.39, 95% CI: 0.24–0.66) and the lower cutoff group (HR = 0.58, 95% CI: 0.39–0.87), although substantial residual heterogeneity remained, particularly in the lower cutoff group (Supplementary Supplementary Table S4).

### 3.8 Pathophysiological mechanisms of PNI in the prognosis of infectious diseases

The following mechanisms provide biological plausibility for the observed association rather than direct mechanistic evidence from the included studies. Severe infection triggers a hypercatabolic and hyperinflammatory response, driving the pathophysiological cascade characteristic of a low PNI (35) (Figure 6).

**Figure 6:**
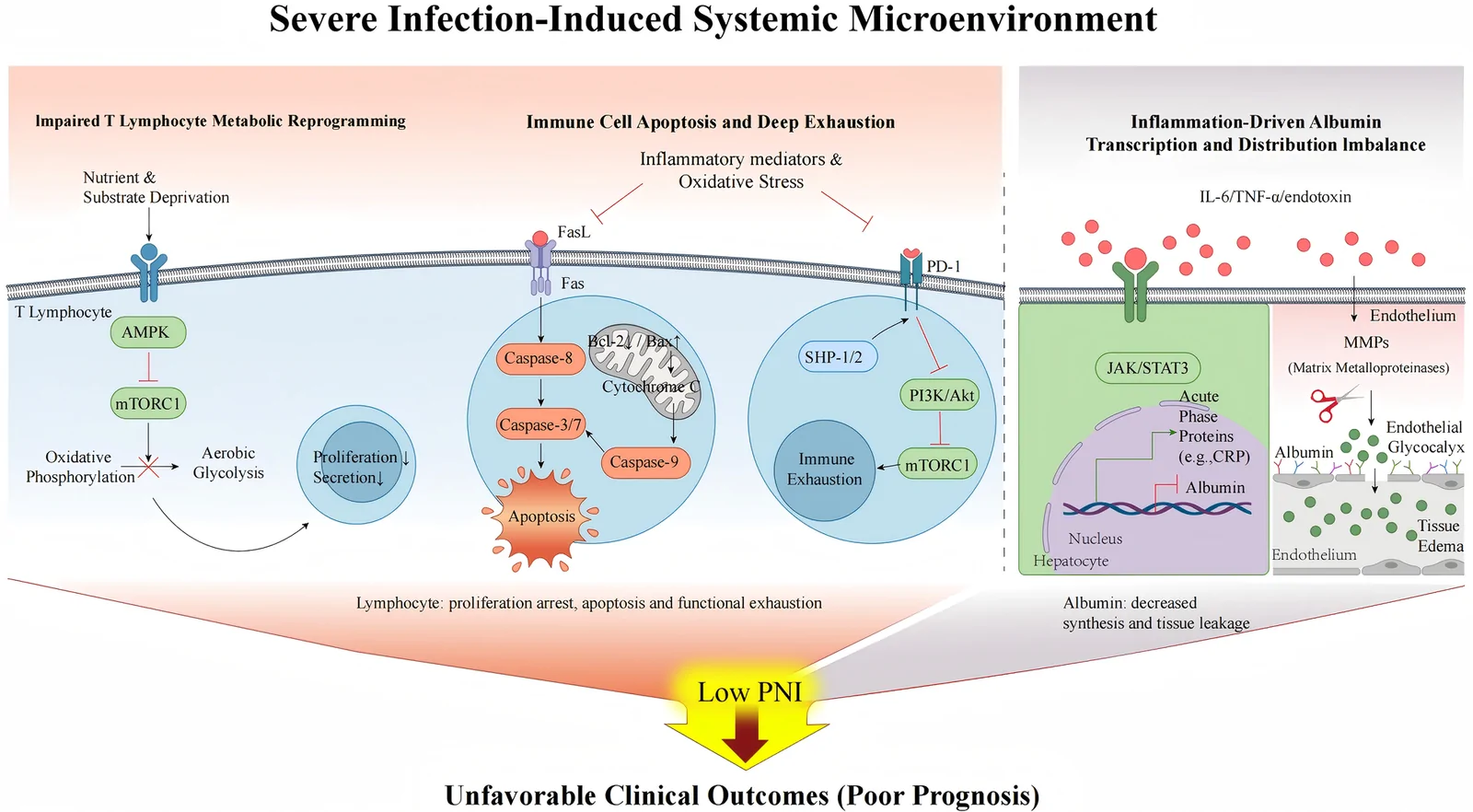
Schematic diagram summarizing the proposed pathophysiological mechanisms of PNI in the prognosis of infectious diseases.

The cascade initiates with a metabolic supply-demand mismatch. High energy requirements coupled with inadequate nutritional intake rapidly deplete intracellular substrates. This acute starvation activates the AMP-activated protein kinase (AMPK) pathway, which inhibits mechanistic target of rapamycin complex 1 (mTORC1) signaling (36, 37), preventing T lymphocytes from shifting to aerobic glycolysis. Deprived of this glycolytic activation, T cells undergo metabolic arrest, halting clonal expansion and effector cytokine secretion (36).

Concurrently, systemic inflammation and oxidative stress trigger massive lymphocyte apoptosis via both the intrinsic mitochondrial and extrinsic death-receptor pathways (38). Surviving T cells enter a state of anergy; prolonged inflammatory stimulation upregulates programmed cell death protein 1 (PD-1), recruiting Src homology 2 domain-containing phosphatase-1/2 (SHP-1/2) phosphatases to disable T-cell receptor-mediated phosphoinositide 3-kinase/Akt (TCR-mediated PI3 K/Akt) signaling (39). Together, metabolic arrest, apoptosis, and functional exhaustion manifest as severe peripheral lymphopenia.

Simultaneously, the inflammatory storm drives albumin depletion. Hepatic IL-6/Janus kinase/signal transducer and activator of transcription 3 (JAK/STAT3) signaling represses albumin synthesis to prioritize the production of acute-phase proteins like C-reactive protein (CRP) (40). This synthetic deficit is exacerbated by peripheral vascular damage: endotoxins and tumor necrosis factor-alpha (TNF-α) activate endothelial matrix metalloproteinases (MMPs), which degrade the glycocalyx (41). The resulting capillary leak syndrome causes extravasation of plasma albumin into the interstitium, leading to diffuse tissue edema.

Ultimately, this synergistic cycle of immunoparalysis and systemic protein depletion provides a sound mechanistic rationale for the high mortality risk observed in critically ill patients with a low baseline PNI.

## 4 Discussion

This meta-analysis of 10 clinical studies comprising 11 independent cohorts suggests that a lower baseline PNI is associated with an increased risk of adverse outcomes, particularly mortality, in patients with sepsis and pneumonia. In contrast, the association was not statistically significant in the tuberculosis subgroup.

The present study offers two primary novelties. First, it represents the first systematic evaluation of PNI specifically within the context of infectious diseases, expanding its prognostic utility beyond the established oncological, cardiovascular, and surgical settings. Second, in contrast to prior meta-analyses that examined biomarkers (e.g., NLR, CONUT, SOFA/APACHE II) within a single infectious disease, our research employs a comprehensive cross-disease framework. By systematically incorporating multiple infectious conditions, these methodological extensions provide a more holistic understanding of PNI and strengthen the current literature.

To further contextualize our findings, we performed a supplementary search of recent meta-analyses evaluating established prognostic metrics in sepsis (42–45) (Table 3). Comparable pooled data for MLR, SII, and NUTRIC/mNUTRIC were not identified; therefore, this comparison was limited to SOFA, APACHE II, CONUT, and NLR and represents an indirect cross-study comparison rather than a head-to-head evaluation within the same cohorts. Importantly, the present meta-analysis did not directly compare the predictive accuracy of PNI with established organ-failure scores such as SOFA or APACHE II. The effect estimates presented in Table 3 were derived from separate studies and meta-analyses and also differed in scoring scales and analytical contrasts, with SOFA/APACHE II estimates generally reflecting a per-unit increase and PNI estimates reflecting categorical comparisons. Therefore, the magnitude of these estimates should not be interpreted as evidence of superior predictive performance of PNI. Rather, the comparison provides contextual information regarding the prognostic associations reported for different host-response and severity indices in sepsis.

**Table 3:** Contextual comparison of prognostic associations reported for PNI and other prognostic indices in sepsis.

Author (year)	Target population	Prognostic predictors	K(N)	OR/HR/AUC (95%CI)	I^2^
Present Study	sepsis	PNI	4 (19867)	HR:0.63（0.44–0.90)	87.9%
Qin et al. (2025) (42)	sepsis	SOFA	5 (3726)	HR:1.19 (1.11–1.27)	72.0%
Qin et al. (2025) (42)	sepsis	APACHE Ⅱ	7 (6439)	HR:1.06 (1.04–1.07)	89.0%
Yang et al. (2026) (43)	sepsis	CONUT	3 (10,196)	OR:1.83 (0.94–3.54)	97.4%
Wu et al. (2024) (44)	sepsis	NLR	11 (10595)	HR:1.69 (1.43–1.99)	77.0%

k, number of included studies; N, total sample size of patients; HR, hazard ratio; OR, odds ratio; AUC, area under the receiver operating characteristic curve; CI, confidence interval; PNI, Prognostic Nutritional Index; SOFA, Sequential Organ Failure Assessment; APACHE II, Acute Physiology and Chronic Health Evaluation II; CONUT, Controlling Nutritional Status; NLR, Neutrophil-to-Lymphocyte Ratio.

The potential clinical relevance of PNI lies primarily in its simplicity and accessibility. Based on only two routinely available parameters—serum albumin and peripheral lymphocyte count—PNI simultaneously captures information related to nutritional and immune status. While combining immune indices (e.g., NLR, MLR, or SII) with nutritional scores (e.g., CONUT or NUTRIC) may provide a more comprehensive assessment, such approaches generally require additional laboratory or clinical variables and the calculation and interpretation of multiple measures. Thus, the potential value of PNI lies in its pragmatic simplicity rather than in a demonstrated superiority over these approaches. Rather than replacing established multidimensional prognostic tools, PNI may serve as an accessible adjunct for early risk assessment, particularly in resource-constrained settings where extensive clinical and laboratory data may not be readily available.

While PNI demonstrated significant prognostic value in sepsis and pneumonia, this association was not statistically significant within the tuberculosis cohort (HR = 0.38, 95% CI: 0.04–3.82). The tuberculosis subgroup estimate was imprecise with very high heterogeneity (I^2^ = 91.2%), based on only three effect estimates with inconsistent endpoints—mortality in two smaller Hu S cohorts versus unfavorable outcomes in the larger Yating Ji study. The latter composite outcome, confounded by drug adherence, multidrug resistance, and malabsorption, may dilute PNI's prognostic signal (32, 33). Additionally, the unreported PNI cutoff in Yating Ji et al. limits comparability. These findings are therefore hypothesis-generating and require further validation. Consequently, the current pooled estimate for tuberculosis should not be interpreted as conclusive evidence against a prognostic role of PNI, but rather as reflecting the limitations of the available data. More importantly, current evidence supports a prognostic association mainly in sepsis and pneumonia, whereas evidence remains insufficient to determine its prognostic value in tuberculosis and other chronic infections.

The substantial between-study heterogeneity observed in the overall analysis (I^2^ = 86.2%) and across disease-specific subgroups (87.9% for sepsis, 71.9% for pneumonia, and 91.2% for tuberculosis) warrants cautious interpretation of the pooled estimates. Although we explored potential sources of heterogeneity through disease-specific subgroup analyses and exploratory meta-regression, no statistically significant moderator was identified, and the relatively small number of available effect estimates limits the ability of these analyses to reliably explain the observed variance. Several clinical and methodological differences may nevertheless contribute to the heterogeneity, including variations in disease severity and clinical setting, patient characteristics and comorbidity profiles, pathogen-related factors, timing of PNI assessment, PNI cutoff definitions, follow-up duration, and treatment or supportive-care practices. Differences in outcome definitions may also be relevant, particularly in the tuberculosis subgroup, where studies assessed either all-cause mortality or unfavorable treatment outcomes. However, these factors represent plausible explanations rather than established determinants of heterogeneity, as most could not be directly evaluated using the available aggregate-level data. Accordingly, the pooled estimates should be interpreted as overall associations across heterogeneous clinical contexts rather than as universally applicable effect sizes.

Furthermore, optimal PNI thresholds appear to be context-dependent, potentially reflecting differences in patient characteristics, disease severity, and clinical setting. Some critical-care and acute infection cohorts used relatively low PNI cutoffs in the low-30 range, whereas Xue et al. reported a higher cutoff of 43.0. The tuberculosis cohort reported by Hu et al. used an intermediate cutoff of 38.7. These differences suggest that a single universally applicable PNI threshold may not be appropriate across infectious diseases and clinical settings. Rather than indicating distinct biological stages of immune-metabolic dysfunction, variation in cutoff values may reflect differences in population characteristics, disease severity, outcome definitions, and treatment context. Prospective studies are therefore needed to determine whether disease-specific or setting-specific PNI thresholds provide more reliable prognostic stratification.

Crucially, the prognostic relevance of baseline PNI may extend beyond the immediate acute phase. While Baek et al. highlighted its association with 1-month mortality in patients experiencing cytokine-storm-related illness, longer-term follow-up studies (28) have also reported associations between diminished PNI and mortality over periods of up to one year. These findings suggest that baseline PNI may capture aspects of host vulnerability that persist beyond the initial acute insult. One possible context for understanding this longer-term vulnerability is Persistent Inflammation, Immunosuppression, and Catabolism Syndrome (PICS), which has been increasingly recognized among critically ill patients who survive the initial hyperinflammatory phase (46–48). Persistent immune dysfunction and catabolic stress in these patients may contribute to susceptibility to secondary infections and adverse longer-term outcomes. However, whether the association between baseline PNI and longer-term mortality reflects persistent immune-nutritional dysfunction or vulnerability to PICS remains uncertain, as the present meta-analysis did not directly assess longitudinal immune trajectories or PICS-related outcomes. Thus, PNI may have prognostic relevance beyond the acute phase, although its ability to capture longer-term host vulnerability requires further investigation.

A low baseline PNI may help identify patients with greater nutritional or immune vulnerability who could be considered for further nutritional assessment. Whether PNI can be used to guide targeted nutritional or immunomodulatory interventions remains unknown and requires prospective and randomized evaluation.

This meta-analysis is constrained by several limitations. Foremost, all the constituent studies employed retrospective cohort designs, which entail inherent selection bias and preclude the complete elimination of confounding variables affecting prognostic outcomes. Despite routine adjustment for baseline covariates via multivariate Cox regression, biomarkers (e.g., procalcitonin, lactate) and pathogen traits like antimicrobial resistance remained inconsistently accounted for across studies. Failure to consistently account for these covariates may have contributed to residual confounding and potentially biased the observed effect estimates. Second, driven by the existing literature, our included cohorts are restricted to a narrow disease spectrum—primarily sepsis, pneumonia, and tuberculosis—which limits the generalizability of our findings to other infectious diseases. Third, relying on a single admission-based PNI overlooks dynamic disease fluctuations. In acute settings, early aggressive fluid resuscitation frequently induces hemodilution, artificially lowering serum albumin. Thus, a depressed baseline PNI may partially reflect iatrogenic fluid shifts rather than pure nutritional depletion; however, because fluid requirements are also related to acute illness severity, fluid-related changes in serum albumin may therefore introduce additional prognostic information that is not specific to nutritional status. Future studies should therefore track PNI kinetics alongside cumulative fluid balance. Finally, the lack of individual participant data precluded stratified analyses, leaving us unable to investigate how the predictive efficacy of PNI varies across different ethnicities, age groups, baseline malnutrition statuses, and specific pathogen types.

Building on these limitations, future research should prioritize large-scale, prospective, multicenter studies using standardized protocols for PNI assessment, outcome definitions, and follow-up. Individual participant data meta-analyses would be particularly valuable for evaluating clinically relevant effect modifiers and improving control of confounding (49). Future prospective cohorts should systematically collect information on age, comorbidities, nutritional status, disease severity, pathogen characteristics, treatment exposures, and the timing of PNI measurement. Longitudinal studies should also investigate whether changes in PNI over time provide additional prognostic information beyond a single baseline measurement (50). Recent studies have suggested that PNI may also be associated with outcomes in Hantavirus-associated hemorrhagic fever and COVID-19 (51, 52); however, evidence in these viral infections remains limited. Future studies should therefore expand the evidence base across a broader spectrum of pathogens, including bacterial, viral, fungal, and parasitic infections, to determine whether the prognostic relevance of PNI is consistent across different infectious etiologies.

Future research should move beyond the search for a single universal PNI cutoff and instead evaluate disease-specific and setting-specific thresholds in adequately powered prospective cohorts. In addition, because PNI reflects immune-nutritional status rather than organ dysfunction, studies should directly evaluate whether integrating PNI with established severity scores such as SOFA and APACHE II improves prognostic discrimination or calibration. Such studies should use appropriate metrics, including time-dependent AUC or C-statistic, calibration measures, and, where appropriate, reclassification and decision-curve analyses. This approach would determine whether PNI provides meaningful incremental information rather than simply reproducing prognostic information already captured by established severity scores.

Finally, mechanistic and interventional studies are needed to determine whether PNI is merely a prognostic marker or whether it can identify patients who may benefit from targeted nutritional or immunomodulatory strategies. Randomized controlled trials would be necessary to establish whether interventions directed at modifiable components of nutritional and immune dysfunction can improve outcomes among patients identified as high risk by a low PNI. Until such evidence becomes available, PNI should be regarded as a potentially useful adjunctive prognostic marker rather than an independent treatment-selection tool.

## 5 Conclusion

Synthesizing data from 11 clinical cohorts, this meta-analysis suggests that a diminished baseline PNI is significantly associated with unfavorable outcomes and higher mortality in infectious diseases (sepsis and pneumonia), whereas its association with tuberculosis remains non-significant. Although PNI may serve as a cost-effective clinical adjunct, substantial heterogeneity, inherent retrospective biases, and a restricted disease scope temper the interpretability and generalizability of the findings. Consequently, further large-scale, multicenter prospective investigations across a broader spectrum of infectious pathologies are warranted to rigorously validate these findings and establish the clinical utility of PNI.

## Data Availability

The original contributions presented in the study are included in the article/Supplementary Material, further inquiries can be directed to the corresponding author.
