# Supplementary material for: Prognostic value of the prognostic nutritional index in sepsis, pneumonia, and tuberculosis: a systematic review and meta-analysis
Source: Front Med (Lausanne). 2026 Sep 21;13:1879397. doi: 10.3389/fmed.2026.1879397 (PMC13634831; doi:10.3389/fmed.2026.1879397)
Supplement: Supplementary file 1 [file Table1.docx]

**TableS1** Literature search strategy.

PubMed (n=393)

#1 "infection*"[Mesh] OR sepsis OR pneumonia OR tuberculosis OR hepatitis
#2 "prognostic nutritional index"[Title/Abstract] OR PNI
#3 #1 AND #2

Web of Science (n=73)

#1 "infection*"[Mesh] OR sepsis OR pneumonia OR tuberculosis OR hepatitis
#2 "prognostic nutritional index"[Title/Abstract] OR PNI
#3 #1 AND #2

Cochrane Library (n=36)

#1 (infection OR sepsis OR pneumonia OR tuberculosis OR hepatitis):ti,ab,kw
#2 ("prognostic nutritional index" OR PNI):ti,ab,kw
#3 #1 AND #2

CNKI (n=85)

#1 SU=(感染 OR 败血症 OR 肺炎 OR 结核 OR 肝炎)
#2 SU=(预后营养指数 OR PNI)
#3 #1 AND #2

Embase (Elsevier) (n=796)

#1 (infection OR sepsis OR pneumonia OR tuberculosis OR hepatitis):ti,ab,kw
#2 ("prognostic nutritional index" OR PNI):ti,ab,kw
#3 #1 AND #2

Note: Literature search was performed from database inception to **June 30, 2026** for all electronic databases, including PubMed, Embase, Web of Science, CNKI, and Cochrane Library.
