# Supplementary material for: Prognostic value of the prognostic nutritional index in sepsis, pneumonia, and tuberculosis: a systematic review and meta-analysis
Source: Front Med (Lausanne). 2026 Sep 21;13:1879397. doi: 10.3389/fmed.2026.1879397 (PMC13634831; doi:10.3389/fmed.2026.1879397)
Supplement: Supplementary file 2 [file Table3.docx]

# Table S3. Univariable and exploratory multivariable meta-regression analyses for sources of heterogeneity.

| **Model_type** | **Moderator** | **k** | **β** | **95% CI** | **P value** | **QM** | **P for moderator** | **τ²** | **Residual I² (%)** | **R² (%)** |
| --- | --- | --- | --- | --- | --- | --- | --- | --- | --- | --- |
| Univariable | PNI cutoff value | 10 | -0.0469 | -0.102 to 0.008 | 0.084 | 3.880 | 0.084 | 0.0653 | 82.1 | 37.4 |
| Univariable | Follow-up duration, months | 11 | -0.0227 | -0.065 to 0.019 | 0.253 | 1.492 | 0.253 | 0.1552 | 91.5 | 0.0 |
| Univariable | Country / region: South Korea vs China | 11 | 0.1859 | -0.987 to 1.359 | 0.724 | 0.361 | 0.708 | 0.1898 | 92.5 | 0.0 |
| Univariable | Country / region: United States vs China | 11 | 0.2679 | -0.4612 to 0.997 | 0.422 | 0.361 | 0.708 | 0.1898 | 92.5 | 0.0 |
| Univariable | NOS score | 11 | 0.1806 | -0.478 to 0.840 | 0.551 | 0.384 | 0.551 | 0.1622 | 93.3 | 0.0 |
| Multivariable exploratory | PNI cutoff value | 10 | -0.0326 | -0.105 to 0.040 | 0.314 | 1.958 | 0.222 | 0.0868 | 81.8 | 16.8 |
| Multivariable exploratory | Follow-up duration, months | 10 | -0.0206 | -0.063 to 0.022 | 0.279 | 1.958 | 0.222 | 0.0868 | 81.8 | 16.8 |
| Multivariable exploratory | NOS score | 10 | 0.1160 | -0.664 to 0.896 | 0.729 | 1.958 | 0.222 | 0.0868 | 81.8 | 16.8 |

Abbreviations: CI, confidence interval; NOS, Newcastle–Ottawa Scale; PNI, prognostic nutritional index; QM, omnibus test statistic for moderators; τ², between-study variance. β represents the change in log (HR) per one-unit increase in the moderator. For country/region, China was used as the reference category. Meta-regression analyses were performed using a mixed-effects model with REML estimation. Given the limited number of included effect estimates, all meta-regression analyses were considered exploratory.
