# Supplementary material for: Prognostic value of the prognostic nutritional index in sepsis, pneumonia, and tuberculosis: a systematic review and meta-analysis
Source: Front Med (Lausanne). 2026 Sep 21;13:1879397. doi: 10.3389/fmed.2026.1879397 (PMC13634831; doi:10.3389/fmed.2026.1879397)
Supplement: Supplementary file 3 [file Table4.docx]

# Table S4. A stratified analysis according to PNI cutoff differences was performed. Studies without reported PNI cutoff were excluded from this stratified analysis.

| **Subgroup** | **k** | **HR** | **CI_low** | **CI_high** | **I2_percent** | **tau2** |
| --- | --- | --- | --- | --- | --- | --- |
| Higher cutoff group (>32.8) | 5 | 0.3941736 | 0.2352436 | 0.6604762 | 63.35501 | 0.09343077 |
| Lower cutoff group (≤32.8) | 5 | 0.5821992 | 0.3913106 | 0.8662069 | 87.83028 | 0.08867952 |

Note: Yating Ji (2025) was excluded because PNI cutoff was not reported.
